# Automatic ICD-10 coding algorithm using an improved longest common subsequence based on semantic similarity

**DOI:** 10.1371/journal.pone.0173410

**Published:** 2017-03-17

**Authors:** YunZhi Chen, HuiJuan Lu, LanJuan Li

**Affiliations:** 1 Zhejiang University School of Medicine, Hangzhou, China; 2 Hangzhou Vocational and Technical College, Hangzhou, China; 3 College of Information Engineering of China Jiliang University, Hangzhou, China; 4 Zhejiang University the First Affiliated Hospital, Hangzhou, China; Huazhong University of Science and Technology, CHINA

## Abstract

ICD-10(International Classification of Diseases 10th revision) is a classification of a disease, symptom, procedure, or injury. Diseases are often described in patients’ medical records with free texts, such as terms, phrases and paraphrases, which differ significantly from those used in ICD-10 classification. This paper presents an improved approach based on the Longest Common Subsequence (LCS) and semantic similarity for automatic Chinese diagnoses, mapping from the disease names given by clinician to the disease names in ICD-10. LCS refers to the longest string that is a subsequence of every member of a given set of strings. The proposed method of improved LCS in this paper can increase the accuracy of processing in Chinese disease mapping.

## Background

Nowadays, the International Classification of Diseases (ICD) codes have been applied in a wide range of fields including statistical analysis of morbidity rate and mortality rate, medical reimbursement and medical record administration [1]. ICD supported by World Health Organization (WHO) means that each disease has a unique code. ICD has been revised and published in a series of editions and the 10th version is ICD-10. In China, ICD-10 codes are usually undertaken by the coder of the hospital’s Medical Record Department according to a doctor’s clinical diagnosis in free text form. After a patient’s clinical treatment, a coder manually assigned an ICD-10 code to that patient’s clinical records document. However, as the coder has to master knowledge in the field of medicine, coding rules and medical terminologies to fulfill the task, manual coding is time-consuming. Meanwhile, manual coding is prone to errors as the human coder has to consider hundreds of possible codes when assigning the correct ICD-10 codes to a document [2].

To solve these problems, we propose a computer aided ICD-10 coding system, which aims to improve the efficiency of working process of coders. There have been a lot of researches on the automated coding of medical texts internationally. An approach for automatic matching of ICD-10 codes to diagnoses was based on multiclass Support Vector Machines (SVM) method [3], but it was used in Bulgarian language. Jon Patrick et al. proposed a machine learning approach associated with bag-of-words model to the task of assigning diseases ICD-9-CM codes to clinical records [4]. Pierre Zweigenbaum and Thomas Lavergne [5] suggested a kind of hybrid methods for ICD-10 coding of death certificates based on SNOMED (systemic nomenclature of medicine) and UMLS (unified medical language source). Damla Arifo˘glu et al. [2] introduced semi-automatic assignment of ICD-10 codes to patient records in Turkish, which proposed a system to offer top k ICD codes for a given patient record using Lucene-based search strategy for each query bag. In top k ICD codes, the best candidate code was selected by a human coder at last. S. Nitsuwat and W. Paoin [6] introduced semi-automated morbidity coding system in Thailand, where the coding system is not a kind of word matching but the ICD-10 ontology in that system is used as a knowledge base in the ICD coding software. Richárd Farkas et al. used a set of hand crafted expert rules to construct an ICD automatic coding systems [7]. However, the feasibility of collecting so many expert rules for thousands of ICD codes is indeed questionable.

Because of the differences in the linguistic features between Chinese and other languages, the approaches for English text or other language text cannot be applied directly to Chinese text, which is characterized by having no delimiter between words and also no apparent morphological markers for them comparing with English text [8]. Only a few researches are oriented at the coding of Chinese medical texts. Wenxin Ning et al. introduced a method for automatic ICD coding of Chinese diagnoses through semantic similarity analysis [9]. With regard to the semantic similarity analysis of Chinese words, a classic method by Liu and Li [10] has been proposed on the basis of HowNet, a Chinese semantic knowledge base. Several subsequent studies [11, 12] are based on this method. Unlike SNOMED and UMLS, HowNet is a domain-independent knowledge base, which doesn’t contain all medical terms, and tends to lead to failure of semantic analysis.

In view of the above situation of semantic similarity, Longest Common Subsequence as a very practical algorithm has been used to measure string similarity comparison in this study, which can describe the similarity between two strings sequence [13]. In our previous research work [14], an experimental hepatitis ontology was created in Chinese, which is made up of many medical terms in the field of hepatitis to emulate HowNet for similarity analysis. On the basis of previous research, this paper proposes an algorithm using the LCS for the automated ICD-10 coding system for clinical disease diagnosis, based on the semantic similarity theory.

The rest of this paper is organized as follows. Section II introduces basic algorithm of LCS and improved algorithm of LCS as well as similarity measure method. The experimental results and discussion are presented in Section III, and finally Section IV concludes the paper with some discussions and ideas for future work.

## Method

### ICD-10 Standard Diagnosis Library

This study is based on the standard diagnostic library (SDL) published and popularized by the Statistical Information Center of the National Health and Family Planning Commission (NHFPC) of the People’s Republic of China. This terminology comprises the standardized names of over 22,000 common diagnoses. Each diagnosis is assigned with a 6-digit ICD-10 code. An ICD-10 code example is shown in Table 1.

**Table 1:** Example of Standard Diagnosis Library.

Standard diagnosis	English translation	code
JIA XING BING DU XING GAN YAN BAN GAN HUN MI	hepatitis A with hepatic coma	B15.000
JI XING JIA XING BING DU XING GAN YAN BAN GAN HUN MI	acute hepatitis A with hepatic coma	B15.001
JI XING ZHONG XING JIA XING BING DU XING GAN YAN BAN GAN HUN MI	acute severe hepatitis A with hepatic coma	B15.002
YA JI XING ZHONG XING JIA XING BING DU XING GAN YAN BAN GAN HUN MI	subacute severe hepatitis A with hepatic coma	B15.003

After a patient’s clinical treatment, clinicians will give a Preliminary Diagnosis of the disease (hereafter referred as PD). Later a coder of ICD will choose a correct ICD code in SDL according to PD that a clinician gave. For example, a doctor's diagnosis is “JIA XING BING DU XING GAN YAN BAN GAN HUN MI” (hepatitis A with hepatic coma) (hereafter referred as A), and then a coder searched for the ICD code in SDL and gave code B15.000 to this diagnosis document at last. However clinicians wrote a diagnosis result in free-text form, they often made a small spelling mistake or made a non-standard diagnosis name or made a similar diagnosis name such as “JIA GAN BAN GAN HUN MI” (hereafter referred as B). The meaning of B is the same as A, so that they should have the same code B15.000. At this situation, if performing the string matching task in Chinese language, there is no correct ICD code to match. In order to deal with these similar Chinese strings, we present a solution using similarity analysis to improve the problem of automatic assignment of ICD codes to patient records in Chinese.

### Basic algorithm description of LCS

The Longest Common Subsequence was firstly proposed by Wagner in 1974 [15]. If a sequence “S” is the subsequence of two or more than two known sequences and meanwhile is the longest among all sequences, then it is the LCS of the known sequence [16, 17].

Hirschberg [18] has provided a solution to this problem with the dynamic programming algorithm. We assume that there are two strings X and Y, of which X = {a0, a1, … am-1} and Y = {b0, b1, … bn-1}. We use a two-dimensional matrix CM*N to store the length of the current LCS in an iterating process. In the matrix, c[i][j] records the length of the LCS from a0 to ai and that from b0 to bj, which provides the solution to the sub-question of the original question. In case i = 0 or j = 0, the empty sequence represents the LCS of ai and bj, which leads to the result of c[i][j] = 0. In other cases, a recursive relation can be established with the semantic similarity taken into account:

$$C[i][j]=\left\{ \begin{array}{l} \;0\;(i=0\;\mathrm{or}\;j=0)\\ \;c[i-1][j-1]+1\;(i,j>0,a_{i}=b_{j)}\\ \max\left\{c[i-1][j],c[i][j-1]\right\}\;(i,j>0,a_{i}\neq b_{j}) \end{array}\right.$$ (1)

Take the disease name mentioned above for an example, “JIA XING BING DU XING GAN YAN BAN GAN HUN MI” (hepatitis A with hepatic coma) referred as A stands for strings X, the other Chinese strings “JIA GAN BAN GAN HUN MI” referred as B stands for strings Y.

### Work flowchart using LCS algorithm

**Fig 1:**
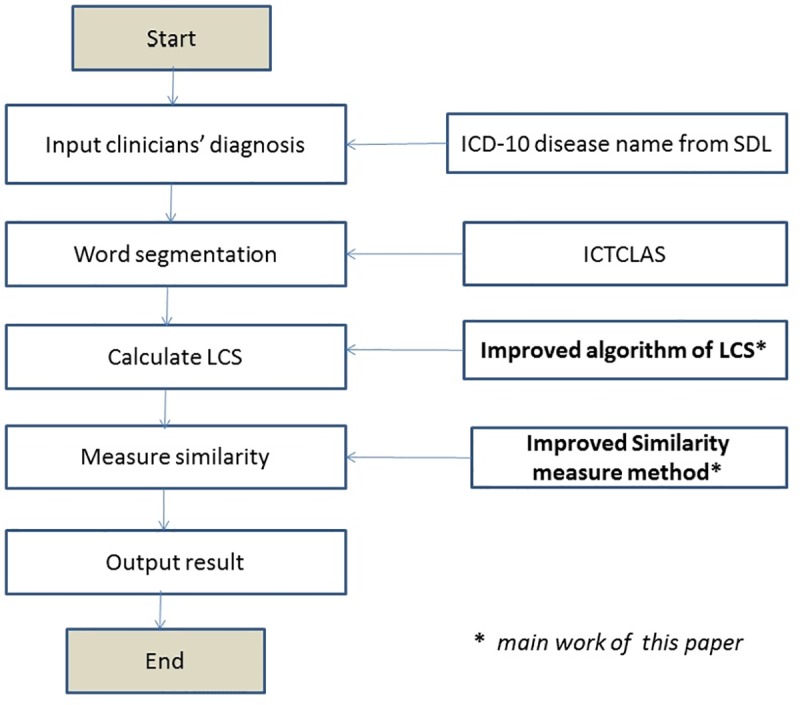
Flowchart of LCS algorithm.

In order to measure similarity between strings X and Y, this paper proposes an automatic code assignment system using LCS algorithm, whose work flowchart is shown in Fig 1.

Step1: Input clinicians’ diagnosis

Firstly, we input the name of the disease diagnosed by the clinician so as to compare with the name of the disease in ICD-10 SDL.

Step2: Word segmentation

Unlike English, there is no delimiter to mark word boundaries and no explicit definition of words in Chinese languages. As for Chinese language processing, the fundamental task is word segmentation, which transforms Chinese character string into words sequence. In this step we use a tool of word segmentation to transform the name of the disease diagnosed by the clinician and the name of the disease in ICD-10 SDL into some Chinese words. Chinese word segmentation in detail was described in the following section.

Step3: Calculate LCS

Thirdly, we calculate the length of LCS between the name of the disease diagnosed and the name of the disease in ICD-10 SDL referring to Eq 1. Eq 1 is an original formula, and this paper proposes a modification on basis of Eq 1. Detailed description will be given in the following section.

Step4: Measure similarity

Then we calculate the semantic similarity based on the improved LCS algorithm proposed in this paper. In order to make full use of the Chinese word sequence information of strings, this paper puts forward a longest common subsequence of text similarity computing method, which is described in the following section of similarity measure.

Step5: Output result

Finally we map the name of the disease diagnosed by the clinician to the name of the disease in ICD-10 SDL based on the semantic similarity and output the ICD-10 coding results.

### Word segmentation

Before calculating semantic similarity, we need to pre-process the medical texts and then to construct the corpus for the convenience of analyzing the diagnosis information. The pre-processing of medical texts mainly includes the following steps:

(1) Chinese Word Segmentation

In the English text, the words are separated by space naturally. However, although characters, sentences and paragraphs can be separated by apparent delimiters, there are, actually, no formal delimiters for words in the Chinese text. In terms of words, segmentation is more complicated and difficult in Chinese rather than in English due to the lack of delimiters. Up to now, a lot of Chinese lexical analyzers have been developed, among which ICTCLAS [19], an analyzer based on the Markov model, is the most popular one. The word segmentation tool applied in this paper is Ansj (http://www.nlpcn.org/demo.jsp) developed by Sun Jiang, in Java version.

(2) Words Filtration

**Fig 2:**
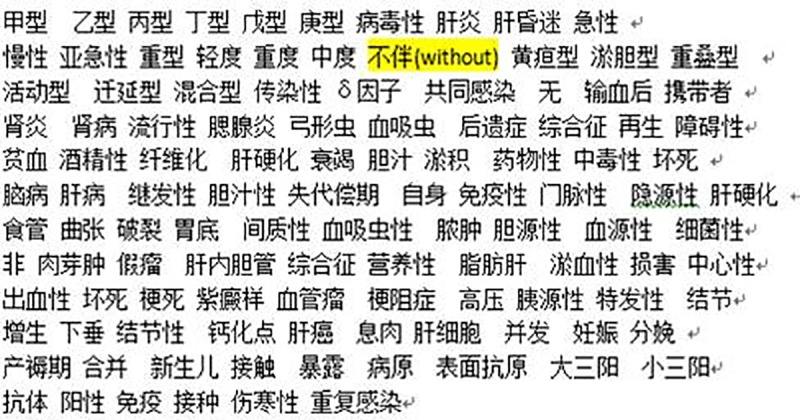
Corpus of Chinese word segmentation of 181 kinds of hepatitis.

In the international classification of diseases, disease names are usually composed of certain medical terms. The purpose of word segmentation is to separate medical terms and remove stop words and meaningless words. Finally, we form a corpus by collecting all the segmentation results. This paper adopts the open source word segmentation tool Ansj to implement this process. The corpus of word segmentation of 181 kinds of hepatitis in ICD-10 SDL is shown in Fig 2.

Taking “JIA XING BING DU XING GAN YAN BAN GAN HUN MI” (hepatitis A with hepatic coma) referred as A as an example, after Chinese word segmentation, string A’s word sequence is “JIA XING” (A) / “BING DU XING GAN YAN” (hepatitis) / “BAN” (with)/ “GAN HUN MI” (hepatic coma). Because “BAN” (with) is a stop word, after word filtration, the final lexical result of string A is likely to be “JIA XING” (A) / “BING DU XING GAN YAN” (hepatitis) / “GAN HUN MI” (hepatic coma). Table 2 shows the result words of 181 kinds of hepatitis and 112 disease names from clinicians. In Table 2, there are 660 words after word segmentation of 181 kinds of hepatitis. 181 kinds of disease names are very similar, which contain a number of repeated keywords, removing stop words and meaningless words. As a result, the corpus only contains 115 words.

**Table 2:** Result words of Chinese word segmentation.

Types of Chinese strings	The number of diseases about hepatitis	The number of words after Word Segmentation	The number of words after word Filtration	segmentation accuracy
Disease names from SDL	181	660	115	96%
Disease names from clinicians	112	532	86	97.1%

Segmentation accuracy in Table 2 is the ratio of the number of correct word to the number of all word after words filtration. Table 2 shows that it is an effective approach to use Ansj as the word segmentation tool to segment Chinese disease names, and its accuracy is above 96%.

### Improved algorithm of LCS

In order to make full use of word frequency information of text itself, this paper proposes a calculation method of the longest common subsequence to measure string similarity.

There exist synonyms or near synonyms in Chinese language. The same meaning can have different names. For example, “hepatitis B” has another name of “serum hepatitis”, “infectious liver disease” is equal to “viral hepatitis”. Considering the diversity of expressions and adding semantic similarity feature to the original LCS algorithm, our study proposes a LCS algorithm that combines the semantic analysis shown in Eq 2.

$$C[i][j]=\left\{ \begin{array}{l} \;0\;(i=0\;or\;j=0)\\ c[i-1][j-1]+1\;(i,j>0,sim[i-1][j-1]>\varepsilon )\\ \;\max\left\{c[i-1][j],c[i][j-1]\right\}\;(i,j>0,a_{i}\neq b_{j}) \end{array}\right.$$ (2)

In Eq 2, sim[i-1][j-1] is the similarity between the i-th subsequence in string A and the similarity of the j-th sequence in string B. If similarity of two feature items is greater than the threshold value ε, namely the two subsequences being matched, the length of LCS dynamic increases one. The previous research mentioned above is that we created disease ontology in Chinese language. Many synonyms or near synonyms in the medical field about hepatitis are included in the ontology. When calculating similarity threshold ε, we can use these synonyms or near synonyms in disease ontology. The improved LCS algorithm in Eq 2 written in Java is given as follows.

***// Returns the length of longest common subsequence of the two strings***

*public static int[][] LCS(String str1*, *String str2) {*

    *int[][] matrix = new int[str1*.*length() + 1][str2*.*length() + 1];*

        *for (int i = 1; i < = str1*.*length(); i++) {*

        *for (int j = 1; j < = str2*.*length(); j++) {*

            *if (sim(str1*.*charAt(i—1)*, *str2*.*charAt(j– 1)> = ε)) {*

                    *matrix[i][j] = matrix[i—1][j—1] + 1;*

            *} else {*

                *matrix[i][j] = (matrix[i—1][j] > = matrix[i][j—1]*? *matrix[i—1][j]*: *matrix[i][j—1]);*

                    *}*

                *}*

            *}*

                *return matrix;*

        *}*

### Measure similarity

Since Hirschberg proposed the algorithm for semantic similarity based on LCS, a lot of researchers have made improvements and optimization on it. Lin Cuiping et al. [20] introduced a common algorithm which explains the ratio of LCS to the comparatively longer length of strings as follows:

$$sim(A,B)=\frac{LCSL}{max\{L(A),L(B)\}}$$ (3)

Twofold LCS [21, 22] (T-LCS, for short) is divided by the sum of the lengths of two strings, shown below:

$$sim(A,B)=\frac{2*LCSL}{L(A)+L(B)}$$ (4)

In Eqs 3 and 4, LCSL is the length value of LCS of two strings, while L(A) and L(B) represent respectively the lengths of feature vectors of strings A and B. Taking “JIA XING BING DU XING GAN YAN BAN GAN HUN MI” (hepatitis A with hepatic coma) (hereafter referred as A) for example again, after Chinese word segmentation, the final lexical result of strings A is “JIA XING” (A) / “BING DU XING GAN YAN” (hepatitis) / “GAN HUN MI” (hepatic coma), so that L(A) is equal to 3.

If the algorithm proposed by Lin Cuiping et al. [20] is applied for calculation, it is assumed that the value obtained will be very small. By adding the weights of length of LCS to improve the accuracy of semantic similarity, we propose an improved method of weighting LCS semantic similarity measure named by W-LCS as follows:

$$Sim(A,B)=\frac{(LCSL+1)*LCSL}{L(A)*LCSL+L(B)}\;L(A)\leq L(B)$$ (5)

Generally speaking, if the LCSL is longer, then two short strings share a higher level of similarity. As it is represented in Eq 5, L(A)) A) (higher level of similarity. As itstrings match that of the longer strings, then the level of similarity increases. Hence we can obtain a bigger similarity value by increasing the proportion of LCSL in the calculation.

## Result and discussion

This paper mainly focuses on the improved LCS algorithm and the similarity results are illustrated in Table 3. According to the SDL that we mentioned above, the longest Chinese disease name is “XING YE HE ZAO XUE QI GUAN DE QI TA JI BING JI SHE JI MIAN YU JI ZHI DE MOU XIE JI BING BING FA YU REN SHEN, FEN MIAN HE CHAN RU QI” (The blood and blood-forming organs of other diseases and involved in the immune mechanism of some diseases complicated by pregnancy, childbirth and the puerperium, ICD code is O99.100). The number of words in this Chinese disease name after word segmentation is 10 which is the maximum L(B) in Table 3. Based on the varied lengths of the input disease name from clinicians and the disease name in ICD-10 SDL, we obtain the LCSL and the algorithm similarity value by three different calculation methods.

**Table 3:** Similarity calculation based on LCS method.

L(A)	L(B)	LCSL	LCS	D-LCS	W-LCS
1	1	1	1.00	1.00	1.00
1	2	1	0.50	0.67	0.67
1	3	1	0.33	0.50	0.50
1	4	1	0.25	0.40	0.40
1	5	1	0.20	0.33	0.33
2	2	2	1.00	1.00	1.00
2	3	2	0.67	0.80	0.86
2	4	2	0.50	0.67	0.75
2	5	2	0.40	0.57	0.67
2	6	2	0.33	0.50	0.60
3	3	3	1.00	1.00	1.00
3	4	3	0.75	0.86	0.92
3	5	3	0.60	0.75	0.86
3	6	3	0.50	0.67	0.80
3	7	3	0.43	0.60	0.75
4	4	4	1.00	1.00	1.00
4	5	4	0.80	0.89	0.95
4	6	4	0.67	0.80	0.91
4	7	4	0.57	0.73	0.87
4	8	4	0.50	0.67	0.83
5	5	5	1.00	1.00	1.00
5	6	5	0.81	0.90	0.92
5	7	5	0.75	0.82	0.90
5	8	5	0.67	0.82	0.87
5	9	5	0.55	0.68	0.84
6	6	6	1.00	1.00	1.00
6	7	6	0.80	0.85	0.91
6	8	6	0.71	0.76	0.83
6	9	6	0.66	0.72	0.78
6	10	6	0.60	0.65	0.70
**Average Similarity**			0.60	0.72	0.78

Table 3 shows that the average similarity value of Eq 5, namely W-LCS is the largest, which verified that our similarity measurement method can enlarge the value of calculation results. The increased value of result is helpful for the following processing of threshold judgment. Comparing with Eqs 3 and 4, there are more threshold values to select for following processing in Eq 5.

**Fig 3:**
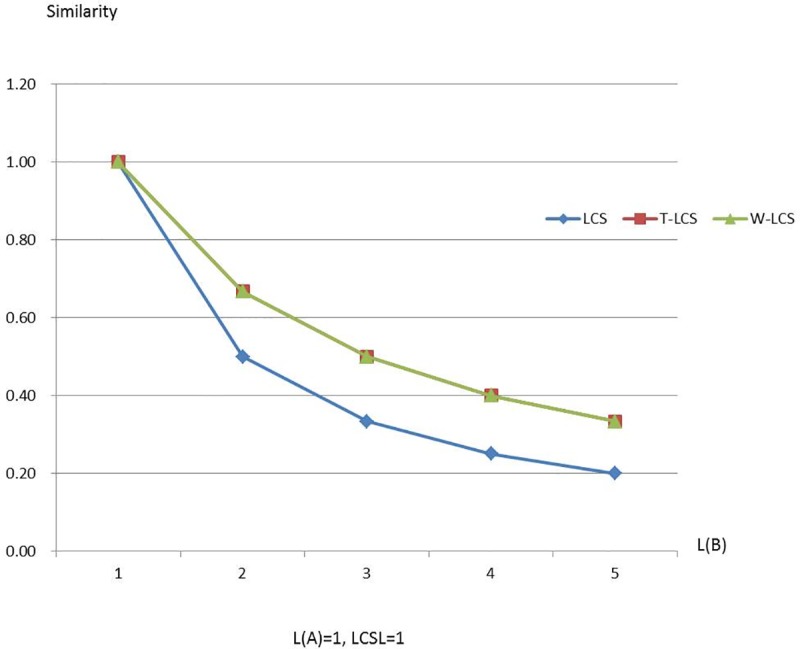
Similarity line chart when L(A) = 1.

**Fig 4:**
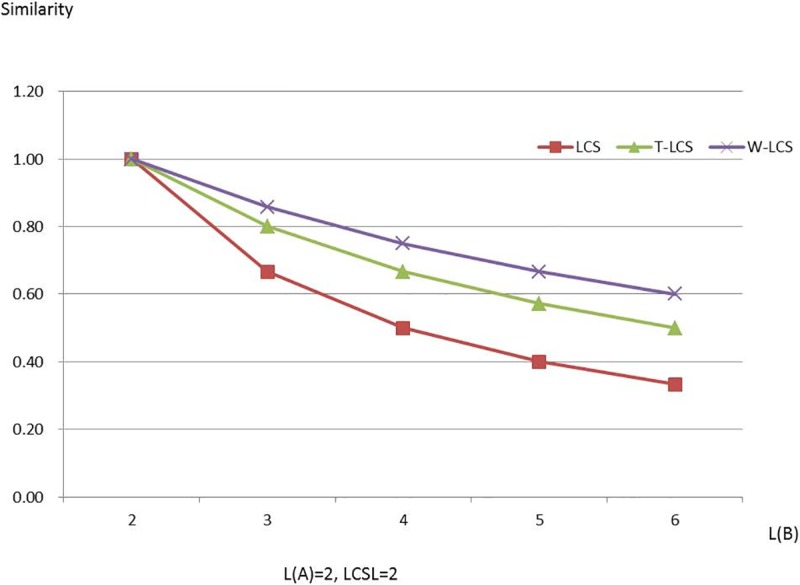
Similarity line chart when L(A) = 2.

**Fig 5:**
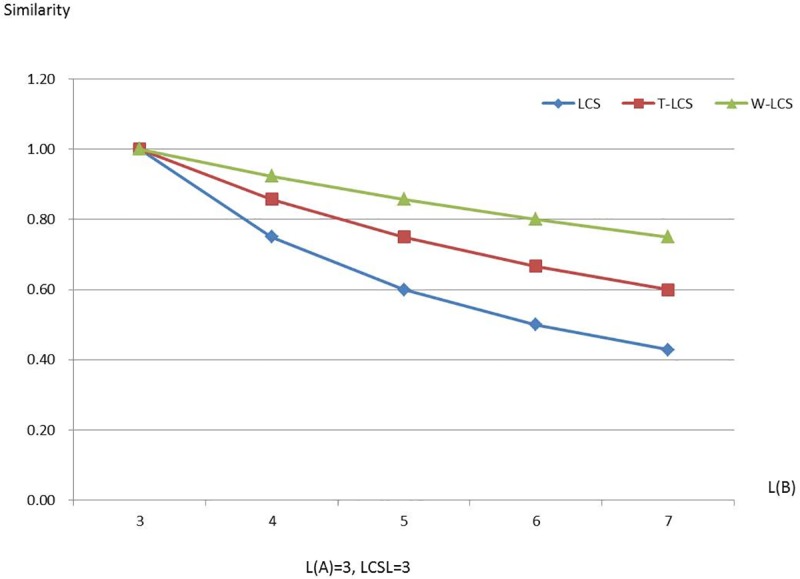
Similarity line chart when L(A) = 3.

**Fig 6:**
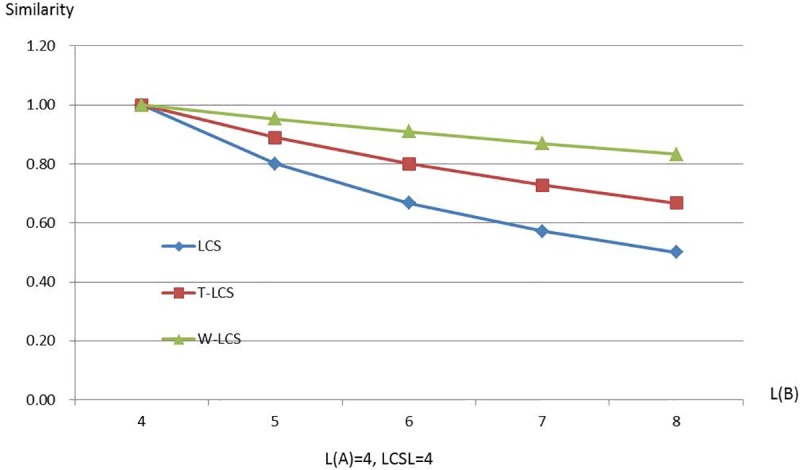
Similarity line chart when L(A) = 4.

**Fig 7:**
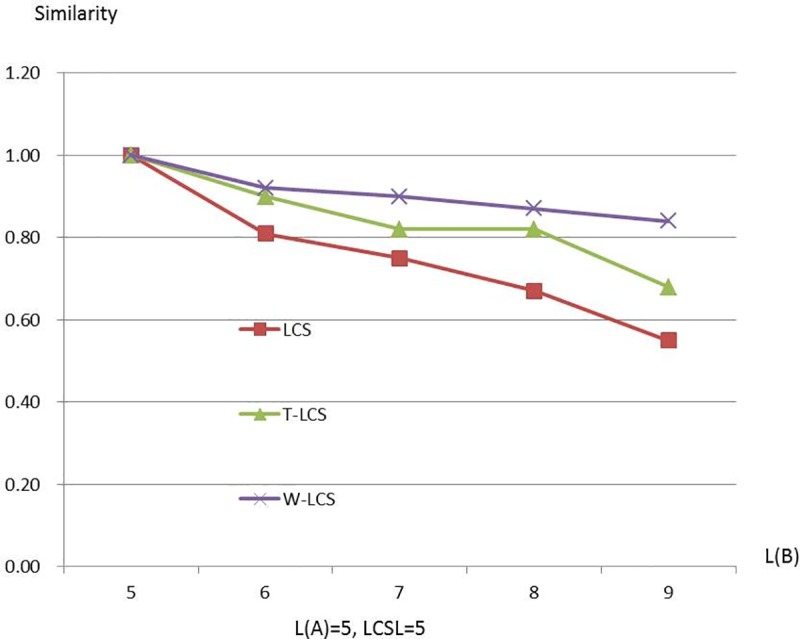
Similarity line chart when L(A) = 5.

**Fig 8:**
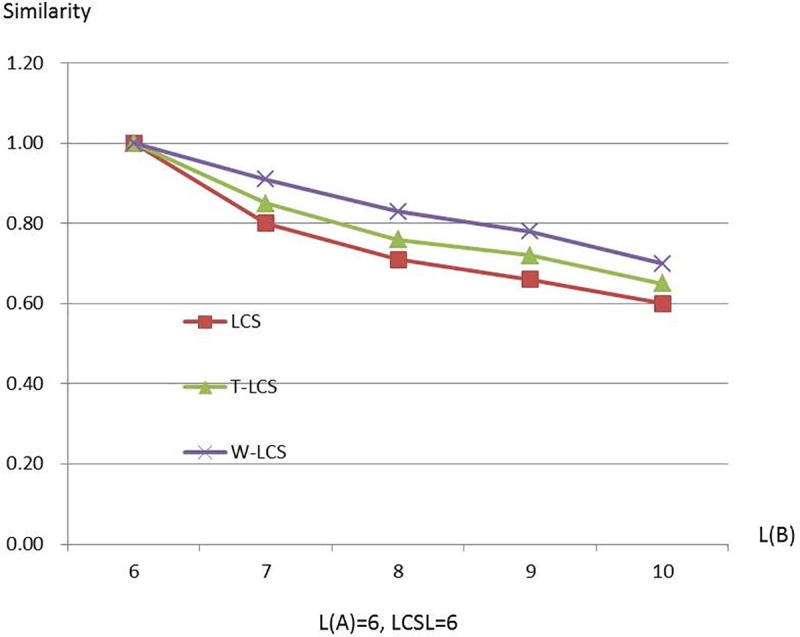
Similarity line chart when L(A) = 6.

Figs 3–8 are line charts demonstrating the similarity changes of different LCS, among which LCS stands for Eq 3, T-LCS for Eq 4, and W-LCS for Eq 5. It is indicated by Table 3 and Figs 3–8 that the similarity value produced by W-LCS algorithm is the biggest among these three similarity algorithms. On the other hand, with the value of LCSL increasing, LCS algorithm is presumed to generate the minimum value of similarity. During the process of accurate matching, the similarity is 1 when the two phrases are exactly the same; otherwise, the similarity is 0. This means that when the two disease names are input for comparison under the same condition, W-LCS algorithm is expected to derive a bigger similarity value. If we are going to search for disease names with the similarity being close to 1 in ICD-10 SDL based on the disease names given by the clinician, W-LCS algorithm can provide more similar options during the search for potential disease names. The result of experiment indicates Eq 5 we proposed is an effective solution by increasing the weight of LCS in formula.

**Fig 9:**
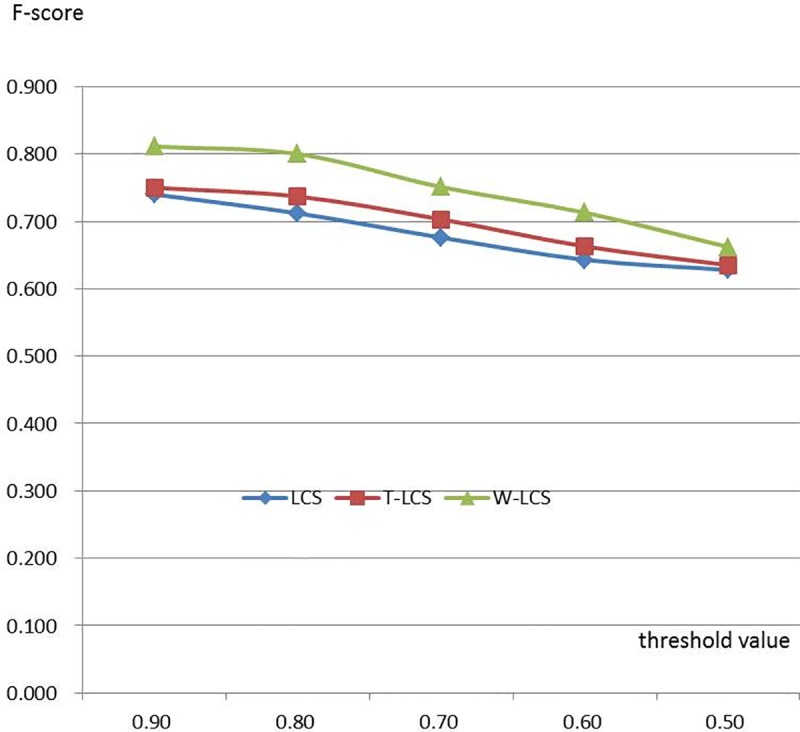
Accuracy analysis chart under similarity threshold (n = 1000).

To evaluate the validity of the proposed coding method, we collected 1000 diagnosis names (about 100 kinds of hepatitis diseases given by clinicians) from the EMRs of our partner hospital as the test set. We set different threshold values. Afterwards, we make a manual check on the accuracy of the algorithms. The accuracy of these three algorithms is shown in Table 4 as well as Fig 9 (which is a line chart of Table 4).

**Table 4:** Accuracy analysis under similarity threshold (F-score).

Threshold value	LCS	T-LCS	W-LCS
0.90	0.740	0.750	0.811
0.80	0.712	0.737	0.800
0.70	0.676	0.703	0.751
0.60	0.643	0.663	0.713
0.50	0.628	0.635	0.662

F-score in the Table 4 is calculated by Eq 6.

$$F-score=\frac{2*precision*recall}{precision+recall}$$ (6)

F-score [23–26] is used in this study as the evaluation metrics. F-score is the harmonic mean of precision and recall. Precision is defined as the ratio of the number of relevant records retrieved (true positives) to the total number of irrelevant and relevant records retrieved (true positives and false positives), which is calculated by Eq 7.

$$precision=\frac{tp}{tp+fp}$$ (7)

Recall shown in Eq 8 is the ratio of the number of relevant records retrieved (true positives) over the total number of relevant records in the test set (true positives and false negatives).

$$recall=\frac{tp}{tp+fn}$$ (8)

Fig 9 shows that the accuracy of the three algorithms increases as threshold value rises. And the accuracy of the W-LCS algorithm improved in this paper ranks the highest among the three algorithms.

Because Chinese language is different from English language, in the process of dealing with stop words, our method is different from general method of words filtration. In English, the word “without” is a preposition, after word segmentation, the word “without” is generally removed from subsequence. However, this paper has a different treatment in the process of Chinese word segmentation, the word “without” cannot be removed from subsequence. Taking “JIA XING BING DU XING GAN YAN BU BAN GAN HUN MI” (hepatitis A without hepatic coma) for example, the final subsequence of strings is “甲型” (A) / “BING DU XING GAN YAN” (hepatitis) / “BU BAN” (without) / “GAN HUN MI” (hepatic coma), namely the word “without” is still retained in subsequence. This is the reason that the word “BU BAN”(without) is in the list of corpus in Fig 2.

To carry out the following experiment, we need a gold standard dataset: a set of clinical diagnoses containing both disease names and codes. As we mentioned above, since doctors fulfill clinical documents in a rush, it may be difficult to assign correct ICD codes for these documents. Moreover, due to the subtle differences between ICD-10 codes, coders frequently disagree on the codes that should be assigned. We constructed a gold standard dataset that two clinic coders chose 2036 clinical documents from the various clinical departments of the partner hospital and these documents contain enough information to be assigned at least one ICD code. If two coders provided the same code, the code is used in the gold standard dataset [23].

In order to explore the semantic similarity that is indispensable in the clinical diagnosis for the task of automatic coding, this paper puts forward a word matching method to compare. In this method, if two words are exactly the same, their similarity is 1, otherwise the similarity is 0. This kind of matching methods is common in the language of English. From Table 5, we can find that, compared with computing semantic similarity of words, the F-score value of word matching method is greatly reduced, only 0.760, which means semantic similarity in clinical diagnosis of automatic coding is indispensable, and also illustrates the process of English language may not be entirely or directly applied to Chinese text. A reference literature from Thailand [6] also confirmed this point of view: ICD-10 coding is not a word matching process, and qualified human ICD coders will never do simple diagnosis word search or browse for the diagnosis term from a list of ICD codes and labels.

**Table 5:** Accuracy comparison of algorithm experiment result.

	precsion	recall	F-score
W-LCS	0.821	0.802	0.811
bigrams	0.819	0.793	0.806
HowNet	0.813	0.756	0.783
word matching	0.781	0.741	0.760

HowNet is a network knowledge base, which describes the relationship between the concepts. HowNet can reflect the relationship between two concepts, including hyponymy, synonymy etc. The version of HowNet used in this paper has 66159 words. However, HowNet is not such a specialty knowledge base of medical domain so that the concepts and their relationships in HowNet cannot be completely applied to the medical field. Currently, there does not exist a medical terminology knowledge base as integrated and open as UMLS or SNOMED–CT in Chinese. W-LCS algorithm has introduced the author’s previous research achievement named Chinese Disease Ontology including 168 kinds of common diseases such as hepatitis, cataract, cholelithiasis and so on, associating with about 1563 ICD-10 codes, which is a project supported by the National Key Technology R&D Program from the Ministry of Science and Technology of the People’s Republic of China. Actually, disease ontology is a disease-oriented specialty knowledge base for medical professionals, whose relationship of hyponymy and synonymous relations etc. are closer to clinical application, which can be verified in Table 5. The F-score in W-LCS algorithm is 0.811, higher than the F-score in HowNet (0.783), which shows the coding accuracy of W-LCS algorithm is higher than that of HowNet.

An n-grams is a contiguous sequence of n items from a given sequence of text [27, 28]. It is based on the assumption that in sequence of text the n-th word depends only on the last n-1 word before it, where bigrams (n = 2) and trigrams (n = 3) are commonly used. If the appearance of one term merely relies on the last one word before it, we call it bigrams. Both W-LCS and bigrams are connected with the order information of Chinese strings. As Table 5 shows, the accuracy of W-LCS algorithm based on Chinese ICD coding is slightly higher than that of the bigrams algorithm. The reasons are given as follows. On the one hand, W-LCS can automatically adjust the length of the matching words according to the test disease name and the real situation of SDL, which is different from the bigrams algorithm that is only based on the one word before it. Taking “HONG MO JIAO MO NEI PI ZONG HE ZHENG”(iridocomeal endothelial syndrome) for example, after words segmentation, the Chinese disease name is divided into “HONG MO JIAO MO” (iridocomeal) /“NEI PI” (endothelial)/ “ZONG HE ZHENG”(syndrome), where there exist Chinese words of two length, of the three length and of four length. W-LCS algorithm using the longest matching, the advantage of doing so is that long words usually describe more precise meanings than short words [29]. However, it still needs further research and experiment for validation. On the other hand, the medical ontology introduced in this paper helps to improve the accuracy, which is verified in the reference literature [30] in which domain ontology is applied in n-gram models to classify Chinese text information.

**Fig 10:**
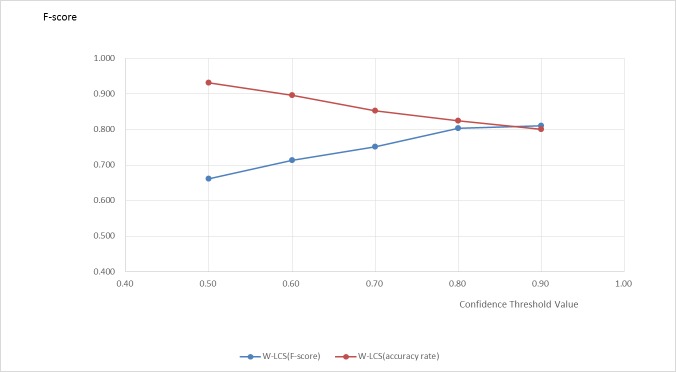
Given threshold of coding accuracy and percentage.

It is difficult for the automatic coding algorithm to achieve 100% accuracy, so in practice, to a certain extent, the algorithm should also have the ability to detect errors. Therefore, this paper brings in an index called Confidence value. When output coding A for a given diagnosis B by clinician, the confidence value is sim (B,TA).The confidence value reflects how much the output coding can describe a given diagnosis, and the reliability of the coding. Fig 10 shows the accuracy and the percentage of the output results when the confidence value of output result is greater than the given threshold in the test set. Obviously, the higher the threshold value is set, the lower the possibility of a greater output confidence value than the threshold value is, and the higher the accuracy of this part of the results is. When the threshold value is 0.803, 82.4% of diagnosis in the test set can be coded higher than the confidence value of this threshold value, and the accuracy of this part of the diagnosis is 0.800 (F-score).This level has reached a high accuracy. Therefore, this part of the coding results can be output directly without manual coding.

The automatic coding algorithm formulated in the practical application is as follows: after input a diagnosis name, there will be a coding result and confidence value. When confidence value is greater than 0.803, the code can be directly output. Otherwise the code should be inspected or recode artificially by coder. According to this process, if confidence value is set to be 0.8, more than 80% of the diagnoses can be coded automatically with high accuracy, and only less than 20% of the diagnoses have to be coded manually by people. If confidence value is set to be 0.5, about 95% of the diagnoses can be coded automatically and directly output. Thus, the workload of human coder can be greatly reduced and the efficiency of coding work can be significantly improved.

Another detail emphasized here again is that the expression sim[i−1][j−1] > *ε* in Eq 2 in our improved LCS algorithm means there is a subsequence comparison between two strings and the result of similarity should be greater than threshold value ε. This is to say, when performing semantic analysis, our study takes subsequence’s synonyms or near synonyms into account. Currently, there does not exist universal Chinese medical terminology knowledge just like UMLS or SNOMED–CT. Our solution to searching synonyms or near synonyms is to implement disease ontology which includes synonyms or near synonyms and was constructed in our previous research containing 168 kinds of common diseases associating with 1563 ICD-10 codes.

## Conclusion

This paper applied the LCS algorithm to the automated ICD-10 coding of Chinese disease names. The LCS algorithm discussed herein takes word sequence into account. After Chinese word segmentation, on the basis of original LCS algorithm, we modified the formula of original LCS. Considering the semantic similarity between two subsequences under the condition of threshold value ε, an improved LCS formula was derived. Moreover, with the introduction of semantic similarity, it is more convenient to apply the ICD coding in clinical practice. On the other hand, we also did a special treatment in similarity calculation. The calculated result obtained by traditional LCS similarity measure was relatively small. Through enhancing the weight of LCS to increase the similarity value, we obtained a new formula of similarity measure. The data acquired from experiments proved that the coding accuracy could be raised by the improved LCS algorithm.

A possible direction for future work will mainly focus on the pre-processing of Chinese and semantic analysis of medical texts. On the one hand, we will optimize this algorithm combined with the bag-of-words method to improve the automatic coding confidence, reducing task of the verification of human coders. On the other hand, we will further study on disease ontology. On the basis of current disease ontology, we will complete the thesaurus of disease synonyms and near synonyms, enhance the accuracy of ICD coding, and apply it to all kinds of common diseases.

## Supporting information

S1 Dataset Corpus of Chinese disease name (181 kinds of hepatitis). (XLS)

S1 Fig Mapping from disease name in Chinese to Chinese phonetic disease name. (TIF)
