# Supplementary figures and images for: Automatic ICD-10 coding algorithm using an improved longest common subsequence based on semantic similarity
Source: PLoS One. 2017 Mar 17;12(3):e0173410. doi: 10.1371/journal.pone.0173410 (PMC5356997; doi:10.1371/journal.pone.0173410)

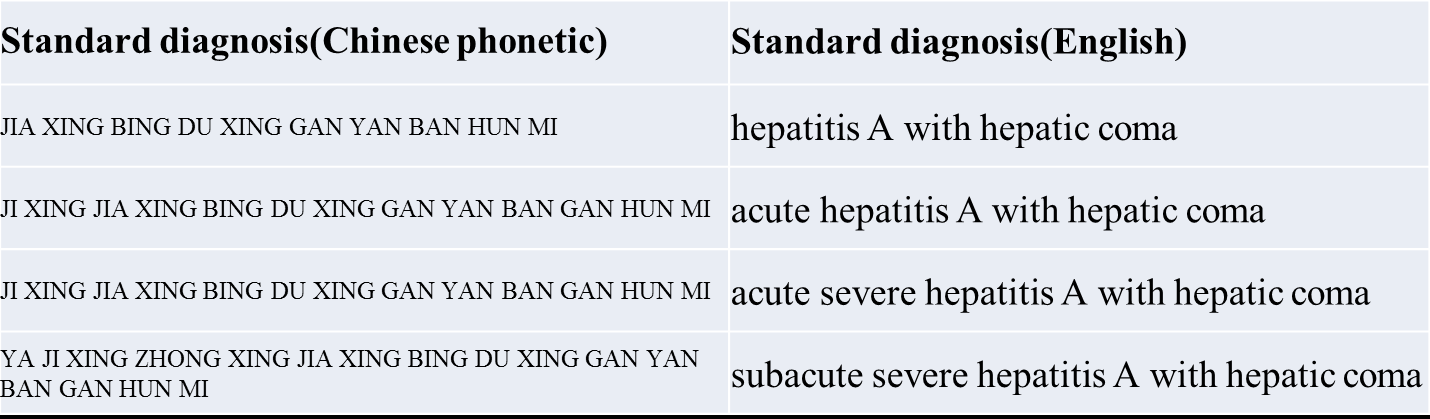

Supplement: S1 Fig — (TIF) [file pone.0173410.s002.tif]
